# Evaluation of Blood Stool Test Utilization for Colorectal Cancer Screening in Georgia, USA

**DOI:** 10.3390/healthcare9050569

**Published:** 2021-05-12

**Authors:** Benjamin E. Ansa, Nicollette Lewis, Zachary Hoffman, Biplab Datta, J. Aaron Johnson

**Affiliations:** 1 Institute of Public and Preventive Health, Augusta University, Augusta, GA 30912, USA; bdatta@augusta.edu (B.D.); jjohnson10@augusta.edu (J.A.J.); 2 Medical College of Georgia, Augusta University, Augusta, GA 30912, USA; nicollette.lewis81@gmail.com; 3 Department of Psychology, Augusta University, Augusta, GA 30912, USA; zhoffma1@outlook.com; 4 Transitions of Augusta, Augusta, GA 30912, USA

**Keywords:** colorectal cancer, screening, blood stool test, FOBT, Georgia, USA

## Abstract

Colorectal cancer (CRC) is the third most prevalent cancer and the second most common cause of cancer-related deaths in the United States (USA). Early screening has been demonstrated to improve clinical outcomes for CRC. Assessing patterns in CRC screening utilization is important for guiding policy and implementing programs for CRC prevention and control. This study examines the trends and sociodemographic factors associated with blood stool test utilization (BSTU) for CRC screening in Georgia, USA. The Behavioral Risk Factor Surveillance System (BRFSS) data were analyzed for Average Annual Percent Change (AAPC) in BSTU between 1997 and 2014 among adults aged 50+ who have had a blood stool test within the past two years, and logistic regression analysis of the 2016 data was performed to identify the associated sociodemographic factors. In Georgia, an overall decrease was observed in BSTU, from 27.8% in 1997 to 16.1% in 2014 (AAPC = −2.6, *p* = 0.023). The decrease in BSTU was less pronounced in Georgia than nationally (from 26.1% in 1997 to 12.8% in 2014 (AAPC = −4.5, *p* < 0.001)). BSTU was significantly associated with black race/ethnicity (Black vs. White (aOR = 1.43, *p* = 0.015)), older age (≥70 vs. 50–59 (aOR = 1.62, *p* = 0.006)), having insurance coverage (no vs. yes (aOR = 0.37 *p* = 0.005)), and lower income (≥USD 50,000 vs. <USD 25,000 (aOR = 0.70 *p* = 0.050)). These findings reveal a decrease over time in BSTU in Georgia, with existing differences between sociodemographic groups. Understanding these patterns helps in directing tailored programs for promoting CRC screening, especially among disadvantaged populations.

## 1. Introduction

Colorectal cancer (CRC), a major clinical and public health concern, is the third most common cancer diagnosed and the second leading cause of cancer-related deaths for both men and women in the United States of America (USA) [1,2]. According to the American Cancer Society, 147,950 new cases and 53,200 deaths from CRC were expected to occur in 2020, and the lifetime risk of developing CRC is about 1 in 23 (4.4%) for men and 1 in 25 (4.1%) for women [3]. Screening for CRC can help identify cancers at an early and potentially curable stage and prevent the development of CRC by allowing the removal of precancerous growths before they become malignant [4,5]. In addition, CRC screening has been shown to improve clinical outcomes such as reducing the incidence and mortality from CRC [6,7,8,9,10].

Several modalities are used to screen for CRC and include blood stool test, sigmoidoscopy, and colonoscopy [9]. In 2019, the American College of Physicians (ACP) recommended that average-risk adults between the ages of 50 and 75 who do not have symptoms should be screened for CRC by a fecal immunochemical test (FIT) or a high sensitivity guaiac-based fecal occult blood test (gFOBT) every two years; colonoscopy every 10 years; flexible sigmoidoscopy every 10 years plus FIT every two years. The ACP advised that average-risk adults younger than 50 years, older than 75 years, or with an estimated life expectancy of less than 10 years should not be screened [11].

Incidence and mortality rates of CRC have declined through the years with previous research finding significant reduction in the overall rate of CRC incidence from 54.5 in 2000 to 38.6 per 100,000 in 2014 with annual percent change (APC) = −2.66 [12]. Likewise, CRC death rates during 2008 through 2017 declined by 3% annually in individuals aged 65 years and older and by 0.6% annually in individuals aged 50 to 64 years [1]. The decline in incidence and mortality rates of CRC has been attributed to screening, and several studies have examined and compared the effectiveness of available CRC screening modalities [13,14,15,16,17,18]. The Fecal Occult Blood Test (FOBT) is synonymous with the blood stool test and reduces the number of deaths from CRC by 15–33% compared to 13–50% for sigmoidoscopy, and 60–75% for colonoscopy [16,17,18,19].

Despite declines in the incidence and mortality rates, CRC remains the number two cause of cancer death in the USA [20,21]. Data from the 2018 National Health Interview Survey (NHIS) found that less than 70% of eligible adults have met the guidelines for CRC screening with differences in screening rates associated with age, race/ethnicity, education, insurance coverage and geographic location [3]. The type of test used to screen for CRC has important implications for compliance with recommended screening intervals [22]. Preferences and rates for the different types of CRC screening tests have been reported across populations and geographic locations by several studies [23,24,25,26,27]. Currently, there are no published studies that have examined the patterns of CRC screening in the state of Georgia, USA. This knowledge may provide an insight into screening preferences among sociodemographic groups and help inform policies and tailored interventions for promoting CRC screening in Georgia. As a first step, the authors of this current project have evaluated blood stool test utilization for CRC screening in Georgia. The specific aims of this study were (1) to determine the prevalence and trends over time of blood stool test utilization for CRC screening, and (2) to examine differences in blood stool test utilization for CRC screening between sociodemographic groups.

## 2. Materials and Methods

### 2.1. Study Participants and Data Source

The study participants were adults from Georgia 50 to 75 years who responded “Yes” or “No” to the question if they had a blood stool test for CRC screening. The 1997 to 2014 and 2016 Behavioral Risk Factor Surveillance System (BRFSS) datasets were analyzed for this study. The BRFSS is a state-based, random-digit-dialed telephone survey of the noninstitutionalized U.S. civilian population aged 18 years or older [28,29]. It is a nationally representative cross-sectional survey that collects data on U.S. residents in all 50 states, the District of Columbia, and three U.S. territories, regarding their health-related risk behaviors, chronic health conditions, and use of preventive services [28]. Currently, the BRFSS survey is sponsored by the Centers for Disease Control and Prevention (CDC) and federal agencies, such as the Health Resources and Services Administration, Administration on Aging, Department of Veterans Affairs, and Substance Abuse and Mental Health Services Administration. The BRFSS datasets can be publicly assessed [28]. Georgia has been part of the system since it was established in 1984 [30]. 

Surveys are conducted through phone interviews (landline and cellphone), and more than 400,000 adult interviews are conducted each year, making it the largest continuously conducted health survey system in the world and a useful tool for addressing and developing health-promotion activities [28,31]. Although conducted in different time periods, the surveys use identical methods for recruitment. Response rates for BRFSS are calculated using standards set by the American Association of Public Opinion Research (AAPOR) Response Rate Formula 4 [32]. The median survey response rate for all states, territories and Washington, DC, in 2016 was 47.0%, and ranged from 30.7% to 65.0% [33]. Response rates for GA included in this analysis had a weighted AAPOR response rate of 48.6 in 2016 [33].

### 2.2. Measures

Trends in blood stool test utilization (BSTU) from 1997 to 2014 were categorized according to sociodemographic variables. Prevalence and odds of BSTU were analyzed using the BRFSS 2016 dataset. The 1997 to 2014 surveys from the online BRFSS Prevalence Data & Data Analysis Tools [34] only asked if respondents have had a blood stool test within the past two years, and the question format changed with the surveys of subsequent years. The survey for subsequent years asked if respondents have had a blood stool test within the past year. Respondents were categorized under sociodemographic variables of sex (male or female); race (non-Hispanic (NH) white, NH black, Hispanic, Asian, American Indian/Pacific Islander, Other); education (less than high school, high school/General Educational Development (GED), more than high school); annual income in United States dollar (USD (less than USD 25,000, USD 25,000–USD 50,000 and more than USD 50,000)); marital status (single relationship (divorced, widowed, separated, never married) and couple relationship (married or a member of an unmarried couple)); healthcare coverage (yes/no). Age in years was categorized as 50–59, 60–64, 65+ for the analysis of the 1997 to 2014 data, and 50–59, 60–69, 70–75 for the 2016 data. The outcome variables were blood stool test within the past two years from 1997 to 2014 (yes/no), and blood stool test within the past year in 2016 (yes/no).

### 2.3. Statistical Analysis

Yearly percentages of BSTU from 1997 to 2014 were calculated for Georgia and USA from the online BRFSS Prevalence Data & Data Analysis Tools [34]. Changes in percentages over time were calculated and expressed as Average Annual Percent Change (AAPC) using the Joinpoint Regression Program (Version 4.5.0.1, Statistical Methodology and Applications Branch, Surveillance Research Program, National Cancer Institute, Bethesda, MD, USA) [35]. The Annual Percentage Change (APC) indicates the utilization rate change at a constant percentage of the rate of the previous year, and is obtained by fitting a least-squares regression line to the natural logarithm of the rates using the calendar year as a regressor variable (the model is linear on the log of the response for calculating annual percentage rate change) [12,36]. The prevalence data were further analyzed by use of joinpoint models, which were aimed at evaluating longitudinal data for a change in trend. An APC was computed for each of those trends by means of generalized linear models, assuming a Poisson distribution. Changes in trend were tested for statistical significance using a Monte Carlo permutation method [12,36]. Average Annual Percentage Change (AAPC) is a summary measure of the trend over a pre-specified fixed interval. It allows us to use a single number to describe the average APCs over a period of multiple years. It is valid even if the joinpoint model indicates that there were changes in trends during those years. It is computed as a weighted average of the APCs from the joinpoint model, with the weights equal to the length of the APC interval.

Descriptive statistics of sociodemographic variables related to BSTU were generated for 2016 using frequencies and proportions. Data were weighted using the iterative proportional fitting weighting method (i.e., raking) to adjust for non-coverage, non-response, and for differences between sample and population characteristics [37]. Raking is a method for adjusting the sampling weights of the sample data based on known population characteristics [38]. Weighted percentages of respondents who reported having had a blood stool test within the past year were calculated for each variable category. A weight is computed for every respondent in a sample, and it is computed by dividing the correct proportion by the observed proportion [37].

Logistic regression analyses were conducted to examine the association between sociodemographic variables and BSTU among study respondents. Data were adjusted for sociodemographic variables (gender, age, race, marital status, education, income and healthcare coverage). Adjusted odds ratios and related 95% confidence intervals were derived from regression analysis. The significance level was set at *p* < 0.05, and all tests were two-sided. Unweighted counts, weighted percentages, and logistic regression analyses were performed using the IBM SPSS version 25 (IBM Corp., Armonk, NY, USA) [39].

### 2.4. Ethical Considerations

BRFSS datasets that are publicly accessible do not contain personally identifiable information. The Centers for Disease Control and Prevention (CDC) ensures that the process of data collection and release are governed by appropriate rules, regulations, and legislative authorizations [29,40].

## 3. Results

### 3.1. Average Annual Percent Change in Blood Stool Test Utilization

**Figure 1:**
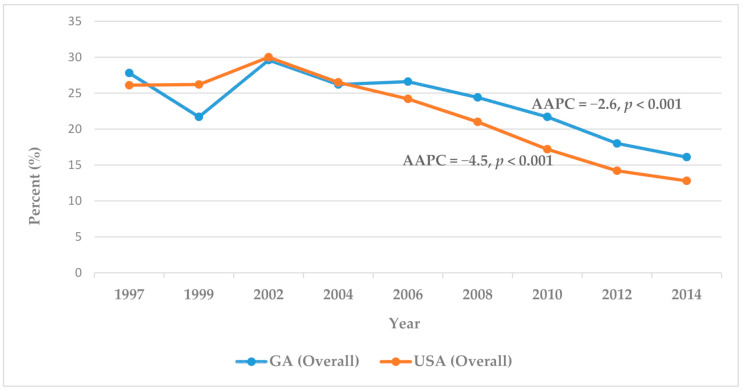
Adults aged 50+ who have had a blood stool test within the past two years.

A comparison between the 1997 to 2014 BRFSS data for Georgia and those of the United States (Figure 1) showed an overall decline in the past two-year BSTU for CRC screening. The nationwide decline was larger than that for Georgia. Between 1997 and 2014, the overall BSTU rate decreased in Georgia from 27.8% to 16.1% (AAPC = −2.6, *p* < 0.001), and nationwide, from 26.1% to 12.8% (AAPC = −4.5, *p* < 0.001). Within this time frame, significant reduction in BSTU was observed among respondents that were White (AAPC = −3.1, *p* < 0.001), female (AAPC = −3.3, *p* < 0.001), 50–59 years (AAPC = −3.6, *p* < 0.001), with high school education (AAPC = −3.1, *p* < 0.001) or college graduate (AAPC = −3.7, *p* < 0.001), and earning USD 50,000 or more annually (AAPC = −4.0, *p* < 0.001) (Table 1). Although the data are not displayed in this report, there was a steady rise in BSTU in Georgia, from 1997 to 2006 (AAPC = 0.8, *p* = 0.7), followed by a sharp decline from 2006 to 2014 (AAPC = −6.4, *p* = 0.1). Likewise, the BSTU rate increased nationwide from 1997 to 2002 (AAPC= 4.0, *p* = 0.2) and declined from 2002 to 2014 (AAPC = −7.0, *p* < 0.001).

### 3.2. Weighted Prevalence of Blood Stool Test Utilization

Analyses of the 2016 BRFSS data examined sociodemographic differences in BSTU for CRC screening. The study participants (*N* = 2583) responded “Yes” or “No” to the question “If you had a blood stool test within the past year” (Table 2). The weighted percentage was calculated for the prevalence of BSTU among the total number of respondents who had a blood stool test within the past year (*n* = 353 (12.8%)). Most of those who had a blood stool test within the past year were Blacks (16.5%), females (13.1%), 70–79 years (17.9%), in a single relationship (13.5%), with less than high school education (15.8%), earning less than USD 25,000 annually (15.1%), and those who had healthcare coverage (13.7%).

### 3.3. Odds of Blood Stool Test Utilization

The odds ratios, corresponding confidence intervals and *p*-values were calculated from logistic regression for the likelihood of BSTU among the study respondents (Table 3). The statistically significant adjusted odds ratios (aOR) were observed among respondents that were black, 70–79 years old, and without healthcare coverage. The respondents that were black and 70–79 years old were more likely to report a past-year BSTU compared to those that were white and 50–59 years old (aOR = 1.429; CI = 1.073, 1.903; *p* = 0.015 and aOR = 1.619; CI = 0.150, 2.277; *p* = 0.006, respectively). Compared to those with healthcare coverage, the respondents that were without healthcare coverage were less likely to report a past-year BSTU (aOR = 0.366; CI = 0.181, 0.741; *p* < 0.005). Although not statistically significant, those earning USD 50,000 or more were less likely to report a past-year BSTU compared to those earning less than USD 25,000 annually (aOR = 0.698; CI = 0.487, 1.000; *p* = 0.050). Gender, education and marital status were not significantly associated with BSTU.

## 4. Discussion

This study provides important information about the prevalence and trends of blood stool test utilization (BSTU) for CRC screening in the state of Georgia, USA. The majority of the study respondents (84% to 86%) have not utilized blood stool test for CRC screening within the past one or two years despite the abundance of evidence that blood stool tests are cheap, readily available, convenient, and reduce the number of deaths from CRC by 15–33%. Overall, the observed decrease in BSTU for CRC screening over time was more pronounced nationwide compared to Georgia, revealing that more Georgia residents are comparatively utilizing blood stool tests. BSTU was significantly associated with older age (70–79), having insurance coverage, lower income, and the Black race/ethnicity.

Similar studies by Klabunde et al. [41] and Seeff et al. [42] reported declines in home BSTU among adults aged 50 to 75 in the USA. In 2005, only 12.0% of the adult population aged 50 to 75 reported BSTU within the past year [43]. BSTU declined during 2000 to 2008 by 6.5 percentage points, occurring among most population subgroups [41]. Additionally, Bandi et al. [23] reported a significant decline of 5.9% points in the rate of BSTU from 2000 to 2008, with significant differences between socioeconomic and racial/ethnic subgroups. These findings are consistent with the results of this current study.

The observed decline in BSTU may be due to the sharp increase in the use of colonoscopy [43], a reflection of care providers’ recommendation for CRC screening. Colonoscopy is considered the gold standard screening tool for the prevention of CRC because it facilitates the detection and removal of precancerous tumors [44,45,46,47]. Therefore, physicians and healthcare organizations recommend colonoscopy to the exclusion of other screening tests, despite access or other challenges to patients in obtaining a colonoscopy [44,48,49,50,51]. In the USA, the use of colonoscopy increased by 8% from 2000 to 2003 [43]. Bandi et al. [23] also observed that those with higher socioeconomic status and non-Hispanic racial groups experienced significant declines in BSTU, while screening by colonoscopy increased, indicating a migration from BSTU to colonoscopy use during the same period. The converse was true for those with lower socioeconomic status. A steady decrease in BSTU and increase in recent colonoscopy rates over a six-year period (2000 to 2005) among all racial, educational, and income groups of Medicare enrollees were also reported by Doubeni et al. [24].

Patient preferences regarding CRC screening may be another reason for the trends observed in this report. Race was the only demographic variable associated with CRC screening test preference in the study by Wolf et al. [48]. Although Wolf et al. observed that Whites were more likely than non-Whites to express a preference for home stool tests, our results show that Blacks were more likely than Whites to report a past-year BSTU, while other racial/ethnic groups were less likely. Studies by DeBourcy et al. [52] and Harden et al. [53] showed that Blacks/African Americans reported a preference for BSTU.

The type of test used to screen for CRC has important implications for compliance with recommended screening intervals. Understanding the differences in CRC screening among populations may guide healthcare providers in promoting the use of more effective methods for CRC screening. Decreasing recommendations for BSTU among healthcare providers in populations with limited access to colonoscopy and without regard to patients’ preferences may result in wider disparities in overall screening rates and require attention from policymakers [25,54,55]. Individual’s preference for CRC screening modality should be taken into consideration when making screening recommendations in order to encourage compliance and meet the national target for screening rates. According to the message by Gupta et al., “The best test is the one that gets done” [55].

### Study Limitations

There are several types of blood stool tests that include the immunochemical fecal occult blood test (iFOBT, or FIT), guaiac fecal occult blood test (gFOBT), and stool DNA test (FIT-DNA). The BRFSS data were not specific about the type of blood stool tests that were utilized by the respondents. Interventions to promote BSTU may be enhanced with the knowledge of respondents’ preferences for specific blood stool tests. Overestimation, underestimation, or misclassification of the results presented may occur from recall bias from self-reporting. Despite these limitations, data from the BRFSS are reliable and generally valid because the content of the survey questions, questionnaire design, data collection, procedures, interviewing techniques, and data processing have been developed to improve data quality [29,56].

## 5. Conclusions

CRC screening rates and compliance are associated with sociodemographic factors and the type of screening test utilized. This study reveals an overall steady decline in BSTU for CRC screening in Georgia that was less pronounced when compared nationally. More Georgia residents are comparatively utilizing blood stool test. In addition, BSTU was associated with being black, older age (70–79), having insurance coverage, and lower income. These findings may have important implications for designing and implementing CRC screening programs locally. Tailored public health and preventive programs that address disparities in the preference of screening modalities are necessary for promoting CRC screening.

## Figures and Tables

**Table 1:** Adults aged 50+ who have had a blood stool test within the past two years in Georgia: 1997–2014 BRFSS data.

Variable	1997 (%)	1999 (%)	2002 (%)	2004 (%)	2006 (%)	2008 (%)	2010 (%)	2012 (%)	2014 (%)	AAPC
Georgia (Overall)	27.8	21.7	29.6	26.2	26.6	24.4	21.7	18.0	16.1	−2.6 *
Gender										
Male	26.3	16.3	30.8	27.0	27.9	23.5	32.1	19.5	15.9	−1.5
Female	29.0	26.2	28.7	25.6	25.5	25.2	20.6	16.8	16.2	−3.3 *
Age (years)										
50–59	22.2	20.0	27.8	24.8	21.6	19.1	17.1	14.6	11.3	−3.6 *
60–64	-	18.5	28.3	29.5	29.2	29.7	24.6	19.7	14.9	−2.0
65+	27.7	24.7	32.2	26.6	31.8	28.6	26.2	21.1	21.6	−1.3
Race										
White	28.8	23.3	31.5	26.4	26.8	24.1	21.6	17.5	15.8	−3.1 *
Black	26.7	15.3	26.8	27.0	28.2	26.5	23.9	19.9	18.0	−0.7
Education										
Less than High School	15.6	16.5	20.2	23.1	19.7	22.0	14.6	15.2	15.2	−0.7
High School/GED	31.3	21.7	28.6	24.3	21.6	24.1	22.0	18.0	15.0	−3.1 *
Some Post High School	33.2	17.5	30.9	27.4	28.3	21.7	23.9	17.8	17.5	−2.4
College Graduate	31.3	30.9	37.4	29.5	32.4	28.2	22.6	20.6	16.2	−3.7 *
Income (USD)										
Less than USD 15,000	17.3	10.3	20.5	22.4	21.3	16.7	14.3	14.5	16.3	0.0
USD 15,000–USD 24,999	16.7	16.1	19.7	25.5	26.4	21.6	20.0	16.1	16.5	0.0
USD 25,000–USD 34,999	29.8	-	28.4	29.3	26.0	23.3	21.7	19.2	17.9	−3.0
USD 35,000–USD 49,999	34.8	14.9	29.5	22.4	28.3	29.1	22.4	19.0	16.1	−1.9
USD 50,000 or more	34.3	27	40.5	28.8	29.2	25.3	22.9	20.5	15.0	−4.0 *
USA (Overall)	26.1	26.2	30	26.5	24.2	21.0	17.2	14.2	12.8	−4.5 *

* Average Annual Percent Change (AAPC) indicates statistical significance (*p* < 0.05).

**Table 2:** Sociodemographics of respondents to the question “if you had a blood stool test within the past year” in Georgia: 2016 BRFSS data.

Variables	Total Study Respondents *N* = 2583	% of Total Study Respondents	Respondents Who Have Had a Blood Stool Test within the Past Year *n* = 353	Weighted % of Respondents Who Have Had a Blood Stool Test within the Past Year *n* = 353 (12.8%)
Gender				
Male	1036	40.1	134	12.4
Female	1547	59.9	219	13.1
Age (years)				
50–59	929	36.0	109	11.6
60–69	1091	42.2	140	12.3
70–79	563	21.8	104	17.9
Race				
White	1696	65.7	215	11.4
Black	662	25.6	111	16.5
Hispanic	79	3.1	8	7.1
Asian	20	0.8	2	11.7
American Indian/Pacific Islander	27	1.0	5	13.6
Other	50	1.9	6	8.7
Not Sure/Refused	49	1.9	6	17.1
Education				
Less than High School	283	11.0	51	15.8
High School/GED	741	28.7	98	11.3
More than High School	1554	60.2	203	12.7
Not Sure/Refused	5	0.2	1	9.0
Annual Income (USD)				
Less than USD 25,000	711	27.5	111	15.1
USD 25,000–USD 50,000	530	20.5	71	11.4
USD 50,000 or more	904	35.0	106	11.5
Not Sure/Refused	438	17.0	65	13.8
Marital Status				
Couple	1447	56.0	191	12.4
Single	1125	43.6	161	13.5
Refused	11	0.4	1	4.3
Healthcare Coverage				
Yes	2383	92.3	342	13.7
No	191	7.4	11	5.3
Not Sure/Refused	9	0.3	-	-

**Table 3:** Odds of blood stool test utilization among study respondents in Georgia: BRFSS 2016.

Variables	Reference	Odds Ratio	95% Confidence Interval	*p*-Value
Gender	Male			
Female		1.019	0.785–1.322	0.889
Age (years)	50–59 years			
60–69		1.057	0.782–1.429	0.717
70–79		1.619	1.150–2.277	0.006
Race	White			
Black		1.429	1.073–1.903	0.015
Hispanic		0.830	0.348–1.977	0.674
Asian		0.555	0.072–4.277	0.572
American Indian/Pacific Islander		1.182	0.336–4.156	0.794
Other		0.772	0.268–2.224	0.632
Education	Less than High School			
High School/GED		0.781	0.505–1.209	0.267
More than High School		0.822	0.534–1.266	0.374
Annual Income (USD)	Less than USD 25,000			
USD 25,000–USD 50,000		0.820	0.581–1.158	0.259
USD 50,000+		0.698	0.487–1.000	0.050
Marital Status	Couple			
Single		0.860	0.646–1.146	0.304
Healthcare Coverage	Yes			
No		0.366	0.181–0.741	<0.005

## Data Availability

BRFSS datasets that were analyzed for this study may be accessed at https://www.cdc.gov/brfss/annual_data/annual_data.htm (accessed on 12 May 2021).
